# Lived Experiences and Long-Term Challenges and Needs of Asian Left Ventricular Assist Device Caregivers

**DOI:** 10.1089/pmr.2021.0001

**Published:** 2021-03-26

**Authors:** Shirlyn Hui Shan Neo, Jasmine Si Min Ku, Jasmine Yun Ting Tan, Sungwon Yoon

**Affiliations:** ^1^ Division of Supportive and Palliative Care, National Cancer Centre Singapore, Singapore, Singapore.; ^2^ Department of Medical Social Services, National Heart Centre Singapore, Singapore, Singapore.; ^3^ Duke-NUS Medical School, Singapore, Singapore.

**Keywords:** caregivers, challenges, left ventricular assist device, needs

## Abstract

***Background:*** Caregivers are essential for improved outcomes in patients living with left ventricular assist device (LVAD). There is a paucity of research on a long-term LVAD caregivers' experiences and burdens.

***Objectives:*** The aim of this study was to explore long-term challenges and needs of LVAD caregivers in the Asian health care setting.

***Design:*** We conducted semistructured interviews with caregivers of patients who were currently or previously living with the LVAD.

***Settings/Subjects:*** Caregivers were recruited from the National Heart Centre Singapore.

***Measurements:*** Interviews were conducted in English and Chinese. All interviews were transcribed verbatim and analyzed based on grounded theory. Chinese interviews were translated to English before transcription.

***Results:*** A multiethnic and multireligious sample of 11 caregivers participated. Median caregiving duration was 45 months. Caregivers described long-term challenges that were multifaceted. Misaligned patient expectations, stigmatization and limited social resources within the family and society affected caregivers' coping. Existing gender roles and spiritual and cultural influences shaped how caregivers appraised, made meaning of caregiving, and assessed support. Long-term caregivers' needs included learning from role models, shifting perspectives, enhancing communication between patient and caregivers, advocacy efforts, and holistic medical care.

***Conclusions:*** Gender roles as well as cultural and spiritual influences affected coping and access to support in long-term Asian LVAD caregivers. Future interventions should consider culturally relevant approaches to improve well-being and quality of life of caregivers.

## Background

Heart failure (HF) is a chronic progressive illness. The left ventricular assist device (LVAD) improves survival and quality of life (QOL) for advanced HF patients.^1^ However, a study that reviewed INTERMACS (Interagency Registry for Mechanically Assisted Circulatory Support) data showed that outcomes are not uniformly good and that up to one-third of patients could die or have serious medical complications such as bleeding or stroke with significant functional impediment, which can have a significant negative impact on QOL.^2^^,^^3^ Patients often experience a total and dramatic change to their lifestyle, hobbies, and activities of living. These include having to learn how to manage a machine, dealing with daily sterile driveline dressings, and frequent attendance of medical appointments.^4^

As living with an LVAD is often too difficult for one person to handle alone, many implanting centers request that the patient must have a suitable caregiver who would be available to assist the patient in these tasks round the clock, and that this caregiver must be committed to care for the patient even before the patient receives the device.^5^ While caregivers were shown to be essential for improved health outcomes in LVAD patients,^6^ limited studies have suggested that LVAD caregivers often find caregiving stressful and filled with uncertainty and are at risk of poor psychological or physical outcomes.^7,8^

However, these studies tended to focus mainly on the time of early LVAD implementation. To date, research on the experience, impact, and challenges of long-term LVAD caregivers is largely sparse.^9^ It is particularly imperative to understand long-term challenges faced by LVAD caregivers in the Asian health care setting as transplants are not readily available and therefore many LVAD patients have to live long term with an LVAD.^10^

Therefore, this study aimed to understand long-term challenges and needs of caregivers of patients living with LVAD in Asian health care setting. By doing so, we seek to develop culturally relevant palliative and supportive interventions to improve long-term caregivers' QOL and well-being The involvement of palliative care in the long-term care of an LVAD patient is recommended in existing guidelines.

## Methods

### Study setting

This study was conducted in the National Heart Centre Singapore (NHCS), a national referral center for heart transplantation and ventricular assist device implantation.^11^ In NHCS, the LVAD patients are cared for by the Mechanical Circulatory Support (MCS) team, consisting of cardiothoracic surgeons, cardiologists, and clinical coordinators. Since 2009, the new generation continuous flow ventricular assist devices have been available in Singapore.^12^ Between 2009 and 2018, 92 patients were implanted with LVAD (65 bridge to transplant [BTT], 26 destination therapy (DT), and 1 bridge to recovery). At the time of this study, 39 patients were still on LVAD support, 28 patients had been transplanted, 2 explanted after recovery, and 23 had died.

### Recruitment

We defined long-term support as caring for an LVAD patient for at least 9 months after implantation.^9^ We recruited caregivers of patients implanted during the period between May 1, 2009, and March 31, 2018, who are currently or previously living with the LVAD. Bereaved caregivers were excluded.

Caregivers were recruited face to face when their loved ones visited the hospital. We adjusted our recruitment iteratively to ensure that we sampled a diverse range of caregivers in terms of age, gender, ethnicity, patient implantation duration, and strategy. Recruitment was performed by a study team member who was not part of the patient's care team.

### Data collection

Information regarding patient LVAD implantation strategy, outcome, and duration of LVAD implantation were collected as well as data on caregiver demographics, the number of hours spent on caregiving per week, and their caregiving roles.

A semistructured interview guide was developed based on the literature^13^^,^^14^ and the study team's research questions. The interview guide was piloted on an LVAD caregiver to test clarity of questions before formal interviews.

Interviews were conducted in a private room in the hospital, in either English or Mandarin. Social workers trained in qualitative research conducted the interviews (Boon Cheng Tan, Eugene Yong Wei Tan, and Genevieve Cheng Sim Wong). Interview time ranged from 1 to 3 hours 18 minutes. All participants gave written informed consent. This study was approved by the SingHealth Centralized Institutional Review Board (Ref. No.: 2018/2013; approved February 20, 2018).

### Data analysis

Interviews were audio-recorded and transcribed verbatim and checked for accuracy before coding. For interviews in Mandarin, transcripts were translated into English. Translated transcripts were reviewed against the original language by the members of the coding team (S.H.S.N., J.Y.T.T., J.S.M.K., and L.S., who are bilingual). Each transcript was examined line by line. All passages pertaining to the research question were coded by the coders independently using a constant comparative approach. Responses were open coded resulting in the formation of code categories. Axial coding was done to look at the inter-relationship of categories. Cross-cutting themes and recurrent patterns were examined until thematic saturation was reached. Discrepancies were resolved through consecutive rounds of discussion. The final codes were agreed on by all members of the research team. Data collection, analysis, and theme generation were ongoing and concurrent processes based on grounded theory.^15^ We adhered to the consolidated criteria for reporting qualitative research criteria (COREQ).^16^

## Results

### Demographics of participants

A total of eleven caregivers were recruited as we reached data saturation at eleven in-depth interviews. Most (90.9%) caregivers were females and all were spouses and main caregivers. Majority of caregivers (10 out of 11) were looking after patients who were still on LVAD support (half were BTT and half were DT). The median duration of caregiving was 45 months (Table 1).

**Table 1.** Characteristics of Caregiver Participants

Characteristics	Caregiver participants (*N* = 11)
Age at insertion of implant (years)	
Mean (SD)	51.3 (9.0)
Age at time of interview (years)	
Mean (SD)	55.5 (8.5)
Gender, *n* (%)	
Male	1 (9.1)
Female	10 (90.9)
Race, *n* (%)	
Chinese	7 (63.6)
Malay	3 (27.3)
Indian	1 (9.1)
Religion, *n* (%)	
Buddhist	3 (27.3)
Taoist	0 (0.0)
Catholic	1 (9.1)
Protestant	3 (27.3)
Hindu	1 (9.1)
Muslim	3 (27.3)
Free thinker	0 (0.0)
Employment status, *n* (%)	
Employed	4 (36.4)
Retired	1 (9.1)
Homemaker	5 (45.5)
Unemployed	1 (9.1)
Duration of caregiving (months)	
Currently on LVAD	
Median (interquartile range)	45.0 (36.8)
Range	9.0–97.0
Mean (SD)	42.7 (19.0)
Transplanted	
Median (interquartile range)	—^a^
Range	—
Mean (SD)	—
No. of hours caregiving per week	
Median (interquartile range)	52.5 (136)
Range	1–168
Caregiving role, *n*	
Physically provide care to the patient	8
Ensure provision of care to the patient	1
Make decisions about treatments	7
Pay for medical expenses	3
Emotional support	11

^a^ There was only one recruited caregiver of a transplanted patient. This patient had previously been on LVAD support for 44.8 months.

LVAD, left ventricular assist device; SD, standard deviation.

### Long-term challenges

All caregivers described the long-term impact of LVAD on their lives. The implantation of LVAD was almost always viewed as a significant milestone or a “second chance” at life and this caused a complete change in different aspects of their lives. In that respect, looking after a patient living with a LVAD meant having to be a constant “learning journey” over time.

The main themes that permeated caregivers' narratives were encountering multifaceted challenges; expectations and ideals of caregiving; and social resources (Table 2).

**Table 2.** Long-Term Challenges and Needs

Main theme	Subthemes	Illustrative quotes
Encountering multifaceted difficulties	Physical Change in roles between patients and caregivers with caregivers having to take on extra heavy physical work	“Because when I see him, so frail, I will tell him ‘you don't do a lot of heavy stuff, I will do it’. I'll be the stronger person now” (CG06, DT).
	Negative impact on caregivers' physical health	“I was thinking, maybe I have not been sleeping well all these nights. That could have caused this. Because I had high blood pressure in the past. When I went to see a doctor, I was still on the high side. 100, 140 or so” (CG 11, DT).
	Loss of sexual intimacy (influence of Asian culture)	“My husband is more old-fashioned. There is no more intimacy because he is afraid. Being a Chinese woman, we are not so highly sexed, so I can understand” (CG 10, DT).
	Loss of sexual intimacy (influence of gender roles)	“The sex life is already totally different. For me it's okay *[to have less sex]* for woman; but for man, he feels like bad” (CG 03, BTT).
	Financial	“Financial will be the only thing irritating the caregiver. You know. You have to provide care and then you have to work for them, you have to do things. No money. You can't provide for the love ones. That is where the caregiver becomes down, I should say” (CG 12, BTT).
	Psychoemotional Anxiety over the lack of medical support (e.g., when travelling)	“I'm afraid that when we go to another country, what will happen to him, and if anything happens, this one is really stressful for the caregiver” (CG 03, BTT).
	Hypervigilance	“Every hour every day I have to be concerned about this thing. Because life is very fragile for us” (CG02, BTT).
	Feeling uncertain about the future	“But I was worried when they gave a time frame for 10 years. I said, ‘I better maximise these 10 years.’ You know, I prayed very hard and in fact I only have another two years left to wait for the heart *[transplant]*” (CG 12, BTT).
	Trauma	“When you see a hospital, you're scared, when you see an ambulance you are also scared. It's like you witnessed a person, who was normal yesterday and then today is like that *[very poorly]*. So it was terrible, it's called trauma” (CG 09, Transplanted).
	Anger due to disagreements over practical tasks and accessing health care	He would ask, ‘Have you cleaned your hands?’ “What?!” I say. ‘I've been handling your wound and there has been nothing wrong with the wound right? do you think it is clean enough?’ (CG 11, DT).
		“Because when I tell him that, you are not supposed to do this. If you have this, we have to see the doctor. He said: “no, no, no, wait. Keep your mouth shut” (CG 12, BTT).
	Emotional coping	“When talking about feelings, there is a problem. Because I don't know who to tell, how to advise him” (CG 03, BTT).
	Getting spiritual support	“My husband said don't tell people. Like for Muslims, when your husband says don't tell that means you must follow. If we tell that means we are opening up the husband's privacy to people. It's a Sin” (CG03, BTT).
	Social Social stigmatization from social circles	“Another thing also how to react to queries from people like for example if suddenly you know a relative across, ‘why do you bring this bag everywhere you go? are you scared you will lose your bag?’ So how do we answer that without hurting… my wife's feelings?” (CG08, BTT).
	Social stigmatization in the community	“But like I said, that is a LVAD, so any time when the alarm it wants to go off, it will go off, wherever you are, in a bus, in a taxi, even shopping centre. So that's the fear that a caregiver has..He's on LVAD then he wants to come out, it's so scary to come out with him” (CG09, transplanted).
Expectations and ideals of caregiving	Ideals Traits	“I have to change to be more patient and to be more gentle, because sometimes, we are human right” (CG 10, DT).
	Duty and obligation out of social, cultural, and religious norms	“This is just me taking care of my family, my younger brother and sister, and now I'm married, and I also have to take care of my family. It's a responsibility” (CG 11, DT). “In our religion, we were taught that once we are entrusted something, we are responsible and have to answer for it. I was handed over the responsibility of my wife.… from her family. I mean I was entrusted and at that point of time her elder brother and her mum handed her over to me so I find that it's already my responsibility, so I am answerable… I mean you are entrusted with a child” (CG 08, BTT).
	Sacrifice	“At first, I don't have any feeling for myself, I only worry about him, and about the two girls and how they are feeling. I must give them encouragement too, to tell them that everything is fine, don't worry. It was only after everything is done, then I realized that I went through all these and it was very difficult” (CG09, transplanted).
	Putting up a front	“I don't show him that I am tired of looking after him. You know, I am tired actually, but I don't show it to him” (CG 12, BTT).
	Expectations Misaligned expectations	“He still thinks he's a boss you see. He had secretaries before, so he still thinks he's a boss and I'm the home secretary” (CG1, DT).
	Dependency of patient	“If I go [*die*] before him, who's going to do the dressing? He has never done it before. Other patients they have done it themselves, but him, he has never even done it once” (CG03, BTT).
Social resources	Role strain within the family Sole caregiver	“Now I hope to let my eldest daughter learn, after some time she may learn how do the dressing. I also want to have an off-day. I have no day off” (CG02, BTT).
	Providing care to other family members	“I need to cook, I need to tuition my daughter. She is Primary 6. I Need to spend time with her PSLE [*primary school leaving examination]*, very crucial. Very tiring” (CG 12, BTT).
	Providing other roles (e.g., financial on top of caregiving)	“Last time I don't have to bother about him, now it's like, before I cook my dinner, I have to dress him first. Otherwise by the time I finish cooking, I'll be very late for my work, so I will have to do dressing for him before I start my own work. It's like some kind of discipline that I need to set” (CG06, DT).
	Lack of understanding from family	“they were scared that he got this. They don't know whether they can touch him or certain things” (Cg 02, BTT).
	Lack of understanding from social network and community service organisations Lack of understanding from community resource organizations	“The Comm Care [*Community Care],* they don't know. ‘What are you? What is this?’ Sometimes we are tired of talking. Tired of explaining” (CG05, BTT).
	Lack of understanding from social circles	“People don't understand us, yeah, sometimes they don't understand. They don't, because it's not in their shoes so they don't understand” (CG 05, BTT).
Shifting perspectives	Role-modelling	“I think it would be good. That you see other patients, and it's very encouraging that everybody is still very happy, living a life very normally, as normally as anybody…” (CG01, DT).
	Reframing Staying positive in mindset	“Even though you are worried, you should try not to let it affect you. Or else how you going to carry on? You still have so many years ahead of you. So you look forward to the good stuff” (CG09, transplanted).
	Making meaning out of challenges	“So whatever my God give me, this is maybe a lesson, or maybe this is a challenge that I have to go through, Maybe I'm the right person can take care of him, so that's why God give me. Or give him to me. Yeah if you're the right person, so the God will say okay this is the right person, just give to him. God won't put person that okay i put this person in this sickness, then I put this person, wah it's really going to be more terrible. So I will respect everything” (CG05, BTT).
Holistic and sustained care	Assessment of needs Supporting the caregivers	“Just talk to them, give them the support. The caregivers need more support than the LVAD” (CG12, BTT).
	Supporting needs beyond the physical	“The clinic only checks through his err records because like, before he goes for his medical check-up, review, then they will ask him to go for some like this check, blood test and all these things. I mean there is no chance of like really talking about if his this one (*pump)* fail, then what will happen” (CG06, DT).
	Management of needs	“So right now, you're used to doing things this way and things are good. But always have in mind that this is permanent you know. This is only going to evolve and it will get worse. Because for young people, it may evolve to something better, they get a heart transplant, they get up, they can even recover. Which is great. But to me the old people, will just get weaker, older, other kind of sickness will come in, other kind of problem will come in. And then it'll become more difficult to cope” (CG01, DT).
Support and advocacy	Support from spouse and family	“Yeah, supporting each other. If … all stress you give to the caregiver, then the caregiver will become mentally also like the same as the sick people when actually we are normal. So the best is we must cooperate together” (CG05, BTT).
	Support from the wider social circle, including employers	“Not only that, I'm working also. I mean my employers sometimes will also, should be also a bit understanding, but especially my immediate la, because my immediate boss… if they are aware of my situation that means they should also like you know. cause I think, cause work-wise if there's no support from your bosses it would be very stressful. Because they don't understand the… the… what I have to go through…” (CG06, DT).
	Support from community/welfare organizations	“If you tell Comm Care *[Community care]* that I have have cancer, they know what is cancer. They know what is a heart attack. But they don't know what is LVAD. And that is why I say sometimes this LVAD should be… should be at least… spread out and some people must know what this is. Sometimes is we… sometimes like even my family, “why you eat with your bag? You take out your bag! I say wait no, cannot because he has an implant.” please, some people also they don't understand how they feelings, they have feelings too. They want to work, but who want to hire them? That is the only the question” (CG05, BTT).

#### Encountering multifaceted challenges

##### Physical

Some caregivers shared that looking after patients whose physical function did not improve after LVAD implantation was challenging. For example, caregivers described that they often had to take on additional physical-related roles on the patient's behalf because patients no longer performed ‘normal’ physical tasks after the implementation of the LVAD. Caregivers recounted that their pre-existing health conditions tended to be exacerbated over time due to chronic lack of rest.

Loss of long-term sexual intimacy or changes to marital life were common challenges for both BTT and DT caregivers. Accounts from participants indicated that the LVAD negatively impacted physical intimacy. “My husband is more old-fashioned. There is no more intimacy because he is afraid” (Caregiver (CG)10,DT). Yet, discussion about sexual issues with partners seemed to be guarded to conform to a culture where female desire is seldom expressed explicitly. Caregivers generally accepted loss of sexual intimacy as part of the LVAD journey.

##### Financial

Concerns about financial instability played a big part in all the caregivers' narratives. The financial concerns were more salient for BTT caregivers, who, besides caregiving, had to work to help pay the financial debts when the patient might not have been able to sustain long-term employment after the LVAD.

##### Psychoemotional

Many caregivers shared that they had lost the hobbies and activities that they enjoyed such as the joy of travel after LVAD implantation, which in turn reinforced the feeling that life after LVAD was considerably “restricted.” This is primarily because of the sense that preparing for the logistics of travelling with the LVAD would be overwhelming or the fear that the patient could be left without medical attention if something unfortunate happened overseas.

Chronic sense of anxiety, insecurity, or heightened vigilance was also common among caregivers. There was a persistent thought that the LVAD would malfunction or the patient would suffer complications due to the illness. Caregivers described the stress of having to constantly monitor the patient to ensure that they could respond as soon as possible when there was anything amiss. For caregivers whose loved ones were waiting for a transplant, the feelings of uncertainty when one would get transplant were not easy to bear. Some caregivers also described how past traumatic experiences related to the patient's acute situation created distressing memories, which they never fully recovered from.

The LVAD also presented itself as a persistent and potential source of conflict between the patient and the caregiver over practical tasks such as dressings or over differing opinions regarding accessing health care resources in times of potential crises. Caregivers were often lost and unsure how to manage their own emotions as well as that of patients when these arguments occurred.

##### Spiritual

Many caregivers described that they turned to their religious faith for support and to make meaning of their caregiving journey. However, some caregivers were hesitant to share information with their spiritual advisors on circumstances surrounding their loved one's LVAD, in respect of the loved one's wish to keep confidential. This was especially evident in culturally traditional Asian families where man is viewed as the breadwinner and head of the household, hence the open sharing of difficulties could be seen as humiliation and loss of pride for the male patient.

##### Social stigmatization

A lack of understanding within the wider social circles of a LVAD often hurts patients and caregivers, thereby creating a sense of embarrassment and avoidance, especially during gatherings or when the LVAD malfunctioned in public places. “So that's the fear that a caregiver has. He's on LVAD then he wants to come out, it's so scary to come out with him” (CG09, transplanted).

#### Expectations and ideals of caregiving

##### Ideals

Both BTT and DT caregivers commonly recounted that at the outset of committing to be a caregiver, they were determined to do their best as a caregiver. They characterized the traits an idealized caregiver embodies as “patient,” “calm,” “accommodating,” and “perseverance.” Caregiving was also described as a duty out of obligation toward a spouse, as per the spiritual and cultural expectations that were inculcated through one's upbringing.

Most participants expressed that they aspired to be an idealized caregiver through constant self-improvement. This means that caregivers would often try to sacrifice and “put the needs of patient and other family members before themselves.” Some mentioned the experience of trying to “put up a front” and keep their own needs to themselves. However, doing so potentially predisposed them to burnout over the long run.

##### Expectations

Caregivers shared some factors that increased their stress. These included misaligned expectations or prolonged dependency from the patient on the caregiver, for example, if the patient was chronically dependent on the caregiver for long-term care and had been unable to manage independently. “If I go [*die*] before him, who's going to do the dressing? He has never done it before” (CG 03, BTT).

#### Social resources

Other challenges that emerged from the interviews included role strain in the family and lack of adequate support from wider community, which often led to an increased sense of isolation and difficulty in coping.

##### Role strain within the family

Role strain in this study was related to how the caregiving roles were delineated within the family, such as (1) having to be the sole caregiver for the patient, (2) providing care to not just the patient, but other members of their family, or (3) performing other noncaregiving duties in the family on top of caregiving (e.g., providing for financial needs). A general lack of understanding of the LVAD from within the typical nuclear and extended family prevented caregivers from receiving much needed support and hands-on assistance long term.

##### Lack of understanding from social network and community service organization

Caregivers also shared that limited understanding within their social circles or from community welfare organizations often hampered the support and services that patients and caregivers could receive.

### Long-term needs

Caregivers shared some possible areas or methods for which they felt further support would be useful. Themes included shifting perspectives; holistic and sustained care; and support and advocacy.

#### Shifting perspectives

##### Role models

Caregivers shared that they would wish for positive role models whom they could learn from, for example, caregivers who have been through the same issues, to give reassurance that one could live a normal life as far as possible over time, despite repeated challenges.

##### Reframing

Learning how to maintain a positive mindset or making positive meaning out of a challenging situation and adversity was important, as it could enable caregivers to look forward to the future with hope. Oftentimes, this could also take on a spiritual lens where some caregivers tried to make meaning out of a difficult situation by trusting that their challenges could be opportunities given from God for their continued growth.

#### Holistic and sustained care

##### Assessment of needs

In terms of support, caregivers also shared their desire that holistic care should not only consider the patients' needs but should also include regular assessments of the caregivers' needs, which was often not the focus of attention during the regular clinic reviews. Caregivers also felt that regular discussions with patients and caregivers about other psychoemotional concerns such as fears about the future should be proactively conducted.

##### Management of needs

Caregivers desired holistic management of their needs, which involved support beyond the physical problems and other aspects of care for the patient and the caregiver. This included planning for or pre-empting future problems such that caregivers would be able to cope better, should those situations occur.

#### Support and advocacy

Caregivers also desired that more people in their social circles such as employers as well as the larger society could be aware of the LVAD and its requirements. In addition, caregivers expressed the need for open communication with their spouse and other family members as they felt that it would allow them to ascribe meaning and focus on their caregiving. “Yeah, supporting each other. If …all stress you give to the caregiver, then the caregiver will become mentally also like the same as the sick people when actually we are normal” (CG05, BTT).

## Discussion

This study explored long-term challenges and needs of caregivers of patients with LVAD within a context of a multiethnic and multireligious Asian community. To our knowledge, this is the first study to describe long-term challenges faced by family caregivers who were involved in care for years after the implementation of LVAD. Our findings suggested that although caregiving challenges may exist at every point along the trajectory of LVAD, long-term caregivers experienced particular challenges that are related to the evolving needs of patients and sociocultural circumstances.

Specifically, this study showed that caregivers' challenges were multifaceted, which resonates with prior literature on caregiving challenges in early/mid phases of LVAD journey.^9^ However, what is different from previous literature is that long-term caregivers' concerns were particularly related to that of prolonged caregiving such as the need for balancing ideals and expectations of long-term caregiving, the sacrifice of normalcy, and the constant grappling within limited familial and social resources to cope with their caregiving burdens. Notably, the traditionally Asian cultural values of “family,” “obligation,” and keeping the LVAD circumstance confidential to “respect the male household head” were also important themes that emerged strongly. In addition, accounts from interviews have illustrated that challenges faced by the BTT and DT caregivers were largely similar; yet, compared to DT caregivers, BTT caregivers were relatively younger and some were at the stage in life where they had a concurrent duty to look after other dependents such as young children.

Overall, our study supports the review by Knight and Sayegh^17^ in that we provided vivid examples of how gender roles and cultural norms impact upon long-term caregivers' appraisal of caregiving stressors and seeking of support. Findings from this study have also highlighted the underlying role of spirituality in caregiving and how caregivers make meaning and see a purpose in caregiving as a responsibility given by God. These findings suggest that cultural tenet may account for long-term caregiving practices in an Asian setting, and therefore, long-term caregivers' challenges should be understood within the larger context of familial, social, cultural, and spiritual aspects of caregiving.

In terms of needs and support, in our previous work on LVAD patients' unmet needs and challenges,^18^ we have shed light on possible interventions. They included (1) having regular assessment of needs and proactive interventions to manage these needs, (2) greater advocacy, and (3) developing a holistic supportive care program to supplement conventional disease-directed care so as to provide an additional layer of support. Likewise, we would suggest that specific interventions be targeted at enhancing caregivers' self-care, improving communication between patients and caregivers, and assisting caregivers in the practical and spiritual aspects of caregiving.

The strengths of our study were that it illuminated the long-term experience of caregiving after an LVAD implantation in a multiethnic and multiracial caregiver sample. Our study highlighted specific cultural nuances about the role, impact, and challenges of long-term caregiving using data derived from rich qualitative interviews. The limitations of this study were that we did not interview other types of caregivers (such as children or siblings), although in the local-regional setting, the predominant type of caregiver is shown to be the spouse caregiver.^19^ Since prior studies identified that spouses can be more distressed than other types of caregivers,^20^ it may be imperative to elucidate the spouse caregivers' experiences of caregiving. Although we did not interview bereaved caregivers, we believe that bereavement support, which is culturally appropriate, should also be considered part of developing a holistic supportive care program.

## Conclusion

In conclusion, our study has highlighted several long-term challenges and needs. We have shown the impact of gender roles and cultural/spiritual influences on caregivers' coping and access to support. Future interventions to improve well-being and QOL of caregivers should consider culturally relevant approaches.

## Abbreviations Used

BTT: bridge to transplant
DT: destination therapy
HF: heart failure
LVAD: left ventricular assist device
MCS: Mechanical Circulatory Support
NHCS: National Heart Centre Singapore
QOL: quality of life
SD: standard deviation
